# Mechanisms of temperature-dependent oxygen absorption/release and appearance of intermediate phase in κ-Ce_2_Zr_2_O_8_: study based on oxygen vacancy formation energy computations†

**DOI:** 10.1039/d2ra02419e

**Published:** 2022-06-06

**Authors:** Hirotoshi Hirai, Ryosuke Jinnouchi

**Affiliations:** Toyota Central R&D Labs, Inc. Yokomichi 41-1 Nagakute Aichi 480-1192 Japan hirotoshih@mosk.tytlabs.co.jp

## Abstract

This study clarified the mechanisms of the temperature-dependent oxygen absorption/release properties and appearance of the intermediate phase for κ-Ce_2_Zr_2_O_8_, which is known to have a high oxygen storage/release capacity (OSC). First-principle computations revealed that the vacancy formation energies depend on the number of vacancies and can be categorized into two groups: low-energy and high-energy. The intermediate phase observed experimentally was assigned to the state after all the oxygen vacancies in the low-energy group were formed. We also found that the mechanism of the improved OSC performance by Ti substitution could be explained in terms of the vacancy formation energies.

## Abstract 2

Oxygen vacancy formation of κ-Ce_2_Zr_2_O_8_.

## Graphical abstract

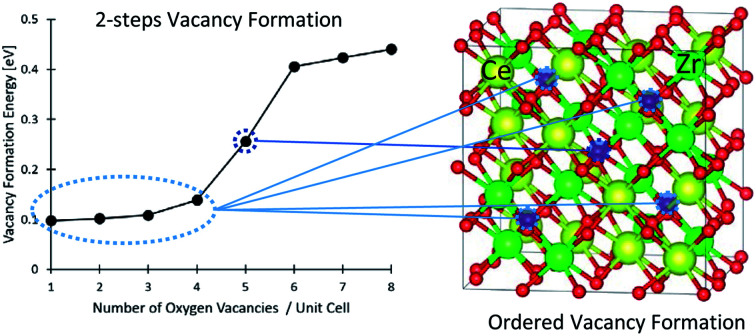

## 1 Introduction

Oxygen storage/release materials have been actively investigated in a wide variety of fields, including environmental protection^1^ and energy conversion.^2^ In these fields, ceria (CeO_2_)-based materials are known to have high oxygen storage/release capacities (OSCs) and are used in industrial applications such as automobile three-way catalysts,^3–5^ water–gas shift reactions,^6^ and solid oxide fuel cells.^7^ In automobile three-way catalysts, ceria-based materials are used as oxygen storage/release materials to adequately control the oxygen concentration on the catalyst surfaces by storing oxygen in an oxidized atmosphere and releasing it under a reduced atmosphere.^3,5,8^ Among the ceria-based materials, cation-ordered Ce_2_Zr_2_O_8_ (κ-Ce_2_Zr_2_O_8_) has been reported to have an outstanding OSC, where almost all the Ce atoms contribute to the redox conversion.^9–11^ The mechanism of the high OSC performance of κ-Ce_2_Zr_2_O_8_ has been studied both experimentally^12,13^ and theoretically.^14,15^ These studies have clarified that oxygen vacancies are formed at oxygen sites coordinated only with Zr atoms, even though the binding strength of Zr^4+^–O^2−^ is usually stronger than that of Ce^4+^–O^2−^. The oxygen vacancy formation energy is known to be dominated by the structural relaxations that occur after the formation of oxygen vacancies. In κ-Ce_2_Zr_2_O_8_, when the Zr^4+^–O^2−^ bond is broken and an oxygen vacancy is formed, the remaining oxygen atoms move toward the vacancy sites as a result of the driving force caused by the differences in the electrostatic forces and ionic radii of Zr and Ce. The oxygen vacancies at the Zr sites are stabilized by this relaxation. Simultaneously, the valency of Zr is retained at 4+ by electron transfer *via* the redox reaction of Ce from 4+ to 3+. This electron transfer prevents the significant movement of Zr atoms, and therefore, the energetically unfavorable lattice distortion is minimized. This latter feature enables the topotactic transformation of κ-Ce_2_Zr_2_O_8_ into Ce_2_Zr_2_O_7_ pyrochlore by maximizing the locality of the structural relaxation.^14^ In theoretical studies, the oxygen vacancy formation energies were examined using density functional theory (DFT) calculations and used to understand the atomic-scale structural changes and oxygen vacancy formation properties.^14,15^ Experimental analyses also clarified the presence of the intermediate phase, β-Ce_2_Zr_2_O_7.5_, in the space group *F*4̄3*m* between the κ-Ce_2_Zr_2_O_8_ (*Fm*3̄*m*) and Ce_2_Zr_2_O_7_ pyrochlore (*Fd*3̄*m*) states.^13,16^ The existence of the intermediate state results in the two-step oxygen absorption/release behavior observed during heating under an oxidized/reduced atmosphere.^16^ Recently, Ti substitution in Ce_0.5_Ti_*x*_Zr_0.5−*x*_O_2_ was found to greatly enhance the OSC performance.^17^ Ce_0.5_Ti_0.1_Zr_0.4_O_2_ was found to have approximately twice the OSC value of κ-Ce_2_Zr_2_O_8_ at a lower temperature (473 K). A bond valence sum (BVS) analysis^18,19^ showed that this OSC enhancement could be explained by the formation of weakly bound oxygen atoms.^17^ However, despite the efforts made in this field, the mechanisms of the appearance of the intermediate phase and the two-step oxygen absorption/release behavior have not yet been fully explained. The mechanism of the OSC enhancement by Ti substitution is also unclear.

In this study, we systematically investigated the formation of oxygen vacancies from a fully oxidized state (κ-Ce_2_Zr_2_O_8_) to a fully reduced state (Ce_2_Zr_2_O_7_ pyrochlore) using DFT, including the Hubbard-U term (DFT+U).^20,21^ The same method was applied to Ti-substituted systems.

The systematic computations of the oxygen vacancy formation energies revealed that they depended on the number of vacancies and could be categorized into two groups: low- and high-energy. The intermediate phase obtained experimentally was assigned to the state after all the oxygen vacancies in the low-energy group were formed. The theoretical conclusion was verified by comparing the simulated X-ray diffraction (XRD) spectra with the experimental results. This new finding reasonably explained the two-step oxygen absorption/release behavior observed in the experiments. The OSC enhancement at low temperatures by Ti substitution was also reasonably explained by the oxygen vacancy formation energy computations.

## 2 Method

### 2.1 First-principle computations

The periodic DFT method was used for the electronic structure computations. The computations were performed using the vienna *ab initio* simulation package (VASP).^22,23^ The generalized gradient approximation (GGA) of the Perdew–Burke–Ernzerhof (PBE) functional^24^ was used to describe the exchange–correlation interaction of electrons. The projector-augmented wave (PAW) method^25^ was used to describe the core–valence interaction. GGA cannot accurately describe the electronic structure of reduced ceria-based materials because of the presence of the self-interaction error.^20,26–28^ Therefore, an on-site Coulomb correction was included using the DFT+U method^29^ to properly incorporate the behavior of Ce 4f electrons. The previous study showed that significant delocalisation because of the self-interaction error that remained for *U* < 5 eV, while the results were converged for *U* ≥ 5 eV where the localisation was found to be independent of the choice of *U*.^20,26^ The similar oxygen vacancy formation energies of CeO_2_ have been reported for *U* greater than 5 eV (3.15, 3.19, 3.20 eV for *U* = 5, 6.1, 6.3 eV, respectively).^15,20,26^ Therefore, the value of *U* was set to 5 eV in this study. Spin-polarized calculations were performed to correctly treat the sub-stoichiometric oxygen. The cut-off energy of the plane wave was set to 400 eV, which has been validated for the CeZrO_2_ system.^15^ The *k*-point sampling was performed using a 2 × 2 × 2 Monkhorst-Pack mesh, except for the calculation of gas-phase oxygen molecules, which was performed using the *Γ* point and a cubic unit cell with 10 Å per side. Both the lattice and atomic positions were fully relaxed to obtain a stable structure. No symmetries were applied to the structural optimization. To investigate the OSC mechanism in detail, Bader charge analysis was performed using the code of Henkelman *et al.*^30^

### 2.2 Model systems

**Fig. 1:**
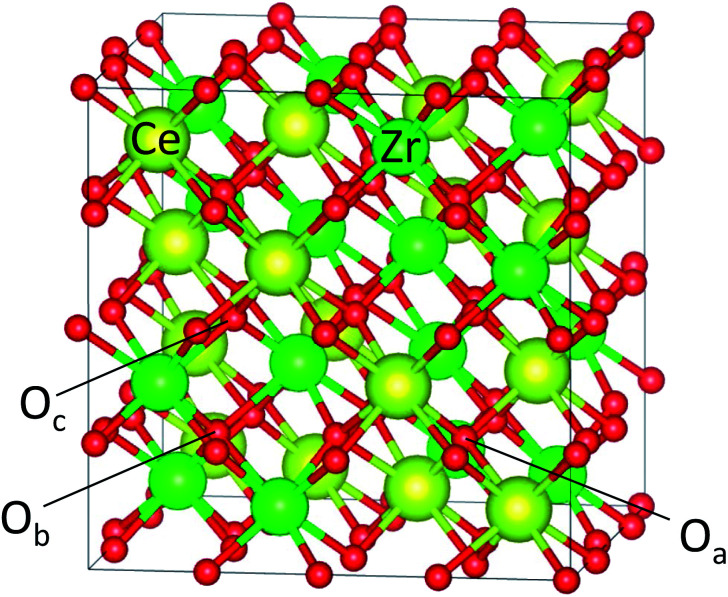
Super cell of Ce_16_Zr_16_O_64_ (96 atoms) for κ-Ce_2_Zr_2_O_8_. The cations (Ce^4+^ and Zr^4+^) were ordered. Examples of three types of oxygen atoms, O_a_, O_b_, and O_c_, are indicated.

The 96 atom super cell (Ce_16_Zr_16_O_64_) shown in Fig. 1 was used for the κ-Ce_2_Zr_2_O_8_ computations. The 64 oxygen atoms in the 96 atom super cell can be classified into three types: 8 O_a_ (coordinated only with Ce), 8 O_b_ (coordinated only with Zr), and 48 O_c_ (coordinated with both Ce and Zr atoms),^10–12^ as shown in Fig. 1. Oxygen vacancies are known to preferentially form at O_b_ sites.^12,14^ When all 8 O_b_ atoms are removed (complete reduction), the remaining 88 atom unit cell represents Ce_2_Zr_2_O_7_ pyrochlore.

To model the Ti-substituted system, we selected a Ce_16_Ti_2_Zr_14_O_64_ system. The Ti cation ratio of this system (6.25 mol%) was close to the composition that exhibited a significant OSC improvement in an experiment (9.7 mol%).^17^ A Ti-substituted system was experimentally confirmed to have a cation-ordered structure.^17^ The preferential vacancy formation sites were considered to be the same as those in the κ-Ce_2_Zr_2_O_8_ system. The formation of oxygen vacancies was expected to be activated by Ti substitution because the Ti–O bond is weaker than the Zr–O bond as a result of the greater electronegativity of Ti^4+^ (the electronegativities estimated by the empirical model^31^ were 1.73 and 1.61 for Ti^4+^ and Zr^4+^, respectively). Therefore, 8 O_b_ sites were chosen as vacancy formation sites. The remaining task was to determine the Ti substitution sites.

**Fig. 2:**
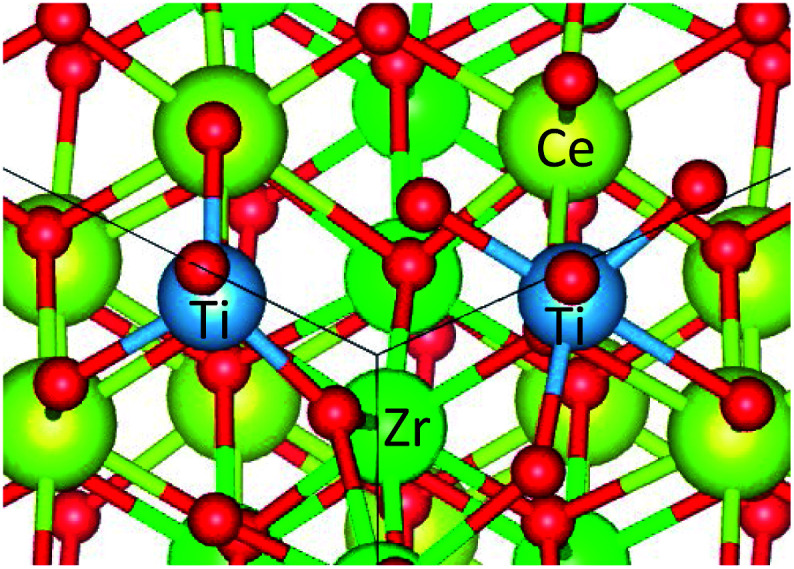
Optimized Ti-substituted structure for Ce_16_Ti_2_Zr_14_O_64_ system.

Several combinations of Zr sites can be replaced by Ti atoms. We examined the calculated total energies of all the symmetrically independent combinations of Ti substitution sites to determine the most stable combination (see ESI† for the calculation result). This examination indicated that in the most stable state, with two Ti substitutions, the two substituted Ti atoms were adjacent to each other (see Fig. 2). Because the ionic radius of Ti^4+^ is smaller than that of Zr^4+^ (Zr^4+^: 0.84 Å, Ti^4+^: 0.74 Å),^32^ the substitution caused a structure distortion around the substituted sites. This distortion could be localized using the configuration shown in Fig. 2 because the induced strain could be cancelled by adjacent substituted Ti atoms. This was considered to be the reason that two substituted Ti atoms energetically preferred the adjacent substitution sites. The oxygen vacancy formation energies of Ce_16_Ti_2_Zr_14_O_64_ were computed using this atomic configuration.

### 2.3 Oxygen vacancy formation energy

The oxygen vacancy formation energy, *E*^f^_v_, relative to the gas-phase O_2_ molecule was computed using the following equation:1
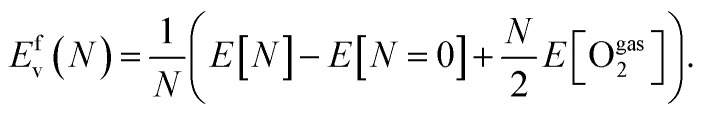
Here, *N* is the number of oxygen vacancies, *E*[*N*] is the energy of the bulk with *N* oxygen vacancies, and *E*[O^gas^_2_] is the energy of the oxygen molecule in vacuum. As previously described, in the bulk energy calculations, the cell volume, shape, and atomic positions were fully relaxed. Unlike previous studies, where *E*^f^_v_(*N*) was computed only for a discrete number of vacancies (*N* = 1, 4, and 8 in ref. 14), we systematically computed *E*^f^_v_(*N*) for all cases (*N* = 1–8), from the fully oxidized state (κ-Ce_16_Zr_16_O_64_) to the fully reduced state (Ce_16_Zr_16_O_56_ pyrochlore).

The oxygen vacancy sites in Ce_16_Zr_16_O_64_ were assumed to be oxygen atoms coordinated only to Zr atoms (O_b_, see Fig. 1). Although all the O_b_ atoms were symmetrically equivalent for *N* = 1, some of the remaining O_b_ atoms became inequivalent after the vacancy formation. In this study, the oxygen vacancy formation energies were examined for all symmetrically inequivalent combinations of O_b_ sites, and the most stable combinations were selected for *E*^f^_v_(*N*). In the case of Ce_16_Ti_2_Zr_14_O_64_, the O_b_ atoms, some of which were coordinated to both Zr and Ti atoms, were not symmetrically equivalent even for *N* = 1. The oxygen vacancy formation energies were computed for all the combination patterns of the O_b_ sites, and the most stable combinations were selected for *E*^f^_v_(*N*).

### 2.4 Symmetry analysis and XRD pattern simulation

The XRD patterns were simulated using the VESTA program^33^ to assign the theoretically obtained structure to the experimentally observed intermediate phase. The FINDSYM program^34^ was used to examine the symmetry of the theoretically obtained crystals and compare them with the space group of the intermediate phase. The tolerance parameters of the program were set as follows: lattice: 0.01, position: 0.1, and rotational moment: 0.1.

## 3 Results and discussion

**Fig. 3:**
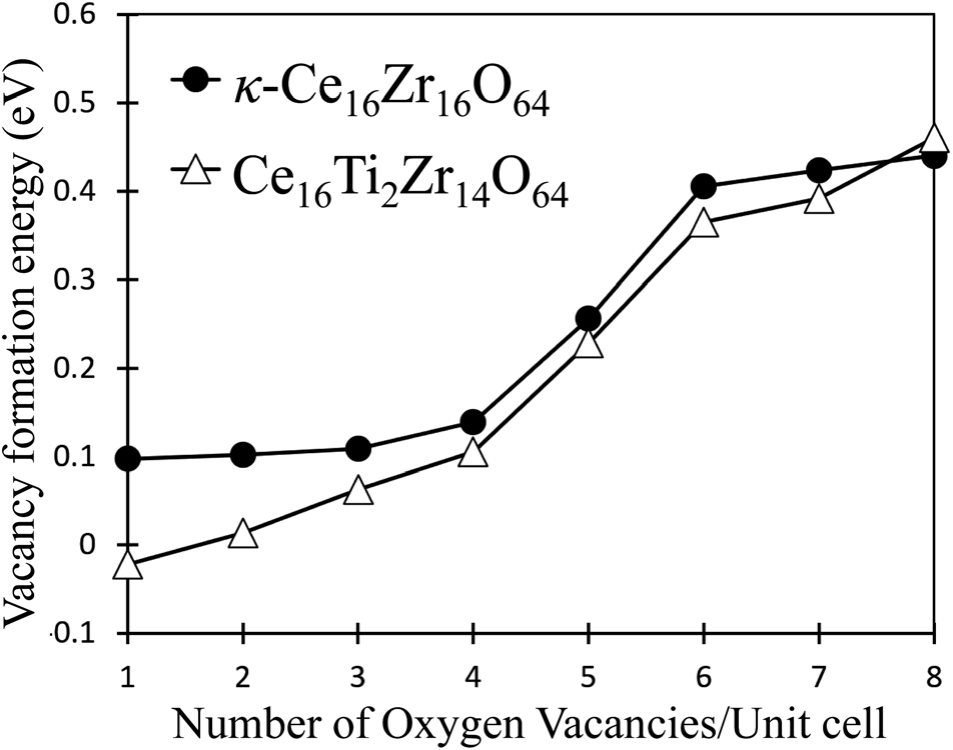
Vacancy formation energies for κ-Ce_16_Zr_16_O_64_ and Ce_16_Ti_2_Zr_14_O_64_ systems.

**Fig. 4:**
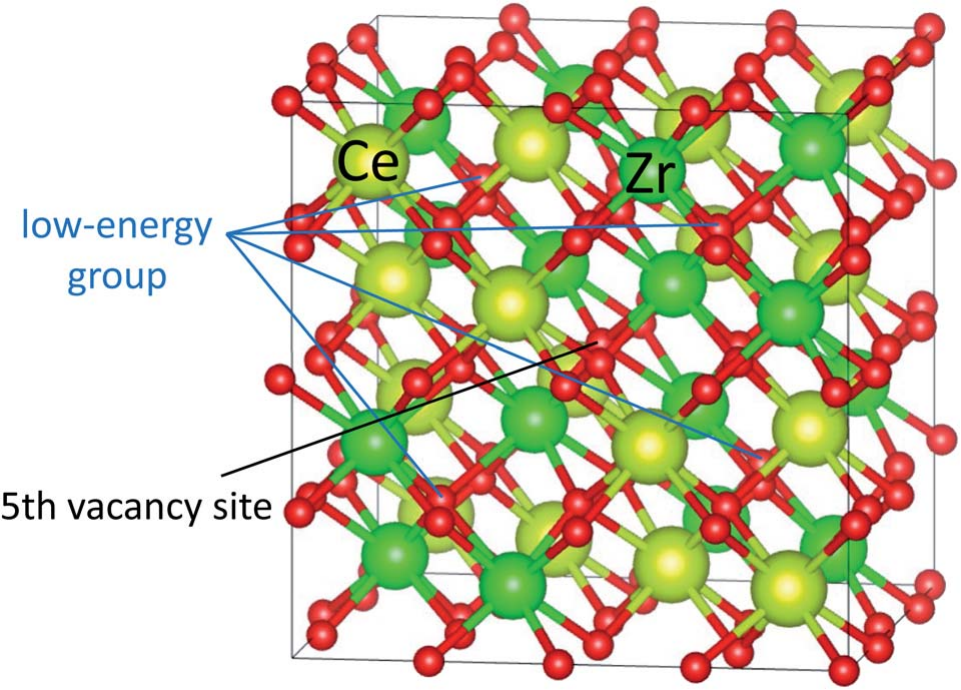
Locations of vacancy sites for low-energy group and 5th vacancy site.

Fig. 3 shows the computed oxygen vacancy formation energies, *E*^f^_v_(*N*), for κ-Ce_16_Zr_16_O_64_ and Ti-substituted Ce_16_Ti_2_Zr_14_O_64_. For both κ-Ce_16_Zr_16_O_64_ and Ti-substituted Ce_16_Ti_2_Zr_14_O_64_, the dependence of the formation energy on *N* appeared to be a sigmoid function, indicating that the *E*^f^_v_(*N*) values could be categorized into two groups: low-energy (first four) and high-energy (last three) groups. The vacancy formation energies of the high-energy group were less than 0.5 eV. These were significantly smaller than those of other materials (*e.g.*, 2.09 eV for tetragonal Ce_2_Zr_2_O_8_ (ref. 14)). This indicated that κ-Ce_2_Zr_2_O_8_ can release oxygen at relatively low temperatures, making it an excellent OSC material. The oxygen vacancy formation energies at *N* = 5 were between those of the two groups. The locations of the low-energy and fifth vacancy sites are shown in Fig. 4. A close examination of the structure indicated that the four vacancy sites in the low-energy group were at locations that maximized their distances. As reported in the literature,^14^ the sacrificial reduction of the surrounding Ce atoms can produce oxygen vacancies with little charge fluctuation of the Zr atoms in the κ-Ce_2_Zr_2_O_8_ system. The maximized distances allow Ce atoms to efficiently influence the vacancy sites *via* the sacrificial reduction mechanism. It should also be noted that the lattice distortions caused by vacancy formation can be minimized by maximizing the distance. Fig. 4 shows that the fifth oxygen vacancy site was located at the center of the four low-energy group sites. This special location was considered to suppress the lattice distortion and make the vacancy formation energy relatively small. However, when additional oxygen vacancies were generated at the high-energy group sites, the symmetry was broken, and the additional vacancy sites had to be adjacent to the existing vacancy sites. In these cases, the distance between vacancy sites became significantly shorter. Therefore, the additional vacancy formations could not benefit from the sacrificial reduction of Ce atoms, and the lattice distortions became significantly large. Accordingly, the two distinct energy groups were attributed to the symmetry of the vacancy formation sites.

**Fig. 5:**
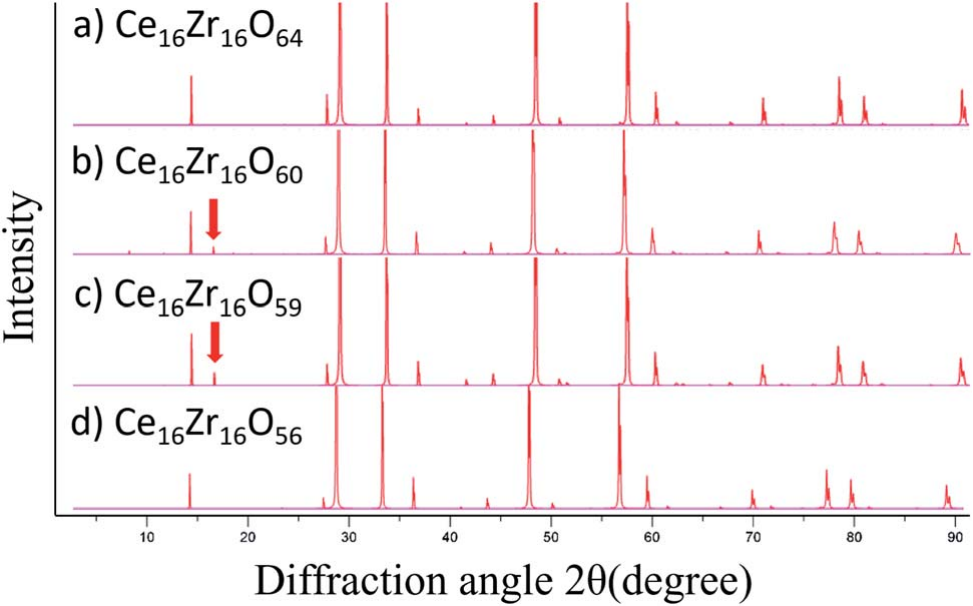
Simulated XRD patterns for (a) Ce_16_Zr_16_O_64_, (b) Ce_16_Zr_16_O_60_, (c) Ce_16_Zr_16_O_59_, and (d) Ce_16_Zr_16_O_56_. The peaks corresponding to a Miller index of (200), which is characteristic of the intermediate phase, are highlighted by arrows.

In the experiment,^13^ a stable intermediate phase with space group *F*4̄3*m* at a composition of Ce_2_Zr_2_O_7.5_ was observed. In this study, the composition of the intermediate phase (Ce_16_Zr_16_O_60_) was assigned to *N* = 4. Therefore, the experimentally observed intermediate phase was considered to be the state after all the oxygen vacancies in the low-energy group were formed. This assignment was supported by the simulated XRD patterns of the structures of *N* = 4 (Ce_16_Zr_16_O_60_), *N* = 0 (κ-Ce_16_Zr_16_O_64_), *N* = 5 (Ce_16_Zr_16_O_59_), and *N* = 8 (Ce_16_Zr_16_O_56_ pyrochlore). The simulated XRD patterns are shown in Fig. 5. The peaks with a Miller index of (200), which are characteristic of the experimentally observed intermediate phase,^13^ are visible in the XRD patterns of Ce_16_Zr_16_O_60_ and Ce_16_Zr_16_O_59_, but do not appear in the XRD patterns of Ce_16_Zr_16_O_56_ and Ce_16_Zr_16_O_64_. For more precise assignments, the space groups were examined for the computed structures of Ce_16_Zr_16_O_60_ and Ce_16_Zr_16_O_59_. The space group of Ce_16_Zr_16_O_60_ was identified as *F*4̄3*m*, which corresponded to the experimentally detected space group of the intermediate phase, whereas that of Ce_16_Zr_16_O_59_ was identified as *P*4̄3*m*. Hence, the experimentally observed intermediate phase was assigned to the state after all the oxygen vacancies were formed at the low-energy group sites. The theoretically obtained formation energy gap between the low- and high-energy groups, as shown in Fig. 3, explains the stability of this intermediate phase.

As shown in Fig. 3, the oxygen vacancy formation energies in the low-energy group were reduced by Ti substitution, whereas no significant change was observed in the high-energy group. This indicated that the release temperatures of the oxygen atoms at the low-energy group sites decreased. In other words, the amount of oxygen released at low temperatures increased. This feature was consistent with experimental results.^17^ Unlike κ-Ce_16_Zr_16_O_64_, the eight oxygen sites, O_b_, in the Ce_16_Ti_2_Zr_14_O_64_ system are not equivalent because of the Ti substitution. Based on the electronegativities of Ti (1.54) and Zr (1.33), we expected that oxygen atoms directly coordinated to Ti would be more easily released than oxygen atoms coordinated only to Zr because oxygen cannot take as many electrons from Ti as Zr. This expectation could be true if vacancies were formed without any structural relaxation, as verified by our preliminary DFT+U calculations on frozen structures. However, when the lattice relaxation was switched on, the vacancy formation energy for the oxygen atoms coordinated only to Zr was smaller than that of Ti. This stemmed from the large strain caused by the removal of oxygen coordinated directly by Ti, which has a smaller ionic radius than Zr.

**Fig. 6:**
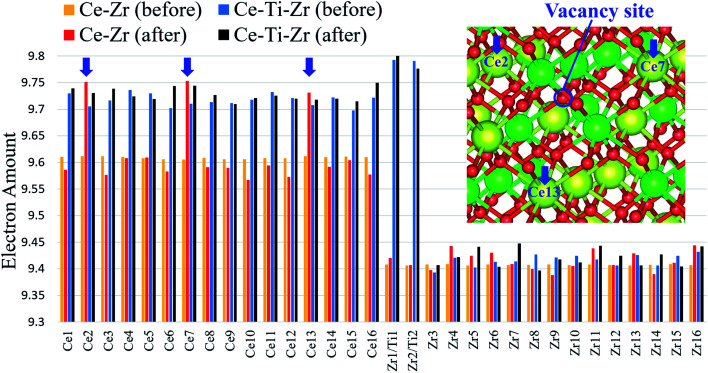
Bader charge amounts for each cation before and after oxygen vacancy formation for κ-Ce_16_Zr_16_O_64_ and Ce_16_Ti_2_Zr_14_O_64_ systems. Three specific reduced Ce atoms are indicated by blue arrows, and their positions are shown in the inset.

Although oxygen vacancies were formed at the Zr sites regardless of the Ti substitution, the oxygen vacancy formation energies were significantly reduced by the Ti substitution. To investigate the mechanism of this reduction, Bader charge analyses were conducted for Ce_16_Ti_2_Zr_14_O_64_ and κ-Ce_16_Zr_16_O_64_. The electron numbers obtained by these Bader charge analyses for all the atoms before and after the first oxygen vacancy formation are compared in Fig. 6. The sacrificial reduction of Ce atoms was observed in the κ-Ce_16_Zr_16_O_64_ system, where the electron numbers of three specific Ce atoms increased when an oxygen vacancy was formed. The reduced Ce sites formed a triangle around the vacancy sites (inset in Fig. 6). However, the electron numbers of Zr atoms, which involved Zr atoms directly coordinating the oxygen vacancy sites, did not change significantly. This result was consistent with that of a previous study,^14^ although two Ce atoms instead of three were reduced during the oxygen vacancy formation in past calculations, where the supercell was fixed during the calculations. This number difference was attributed to the relaxation of the supercell: two Ce atoms were reduced when the supercell was fixed in our preliminary calculations. In contrast, in Ce_16_Ti_2_Zr_14_O_64_ systems, the electron amounts for the Ce atoms were already increased with the Ti substitution before the vacancy formation, as shown in Fig. 6. These electrons were confirmed to originate from the oxygen atoms (the Bader charge for the oxygen atoms was decreased by the Ti substitution). However, the number of electrons in the Zr atoms was not affected. The decrease in the number of oxygen electrons resulted in a decrease in the strength of the ionic bonds. This was considered to be the cause of the decrease in the oxygen vacancy formation energies.

## 4 Conclusion

In this study, we systematically investigated the formation of oxygen vacancies from the fully oxidized state (κ-Ce_2_Zr_2_O_8_) to the fully reduced state (Ce_2_Zr_2_O_7_ pyrochlore) using first-principles computations based on the DFT+U method. Systematic computations of the oxygen vacancy formation energies of cation-ordered Ce_2_Zr_2_O_8_ revealed that the arrangement of vacancy sites was determined so that the distance between vacancy sites was as large as possible, and the symmetry was as high as possible. This ordered arrangement minimized the lattice distortion and maximized the benefit of Ce reduction during the vacancy formation. This feature resulted in the appearance of two oxygen vacancy formation energy groups: low- and high-energy groups. The intermediate phase obtained experimentally was assigned to the state after all the oxygen vacancies in the low-energy group were formed on the basis of the simulated XRD spectra. The theoretically obtained oxygen vacancy energies explained the two-step oxygen absorption/release behavior observed experimentally during heating under oxidized and reduced atmospheres. In addition, this ordered vacancy formation was considered to be one of the reasons for the possibility of topotactic transformation between κ-Ce_2_Zr_2_O_8_ and Ce_2_Zr_2_O_7_ pyrochlore. The OSC enhancement by Ti substitution at lower temperatures was also reproduced by the theoretical oxygen vacancy formation energies. The charge transfer from oxygen atoms to Ce atoms caused by the Ti substitution was considered to be the main factor for the improved OSC.

## Conflicts of interest

The authors declare that they have no conflicts of interest.

## Supplementary Material

RA-012-D2RA02419E-s001
