# Supplementary material for: Mechanisms of temperature-dependent oxygen absorption/release and appearance of intermediate phase in κ-Ce2Zr2O8: study based on oxygen vacancy formation energy computations
Source: RSC Adv. 2022 Jun 6;12(26):16717–22. doi: 10.1039/d2ra02419e (PMC9169551; doi:10.1039/d2ra02419e)
Supplement: RA-012-D2RA02419E-s001 [file RA-012-D2RA02419E-s001.pdf]

Electronic Supplementary Material (ESI) for RSC Advances.
This journal is © The Royal Society of Chemistry 2022

Supporting Information for "Mechanisms of temperature-dependent oxygen absorption/release and appearance of intermediate phase in κ-$Ce_2Zr_2O_8$: study based on oxygen vacancy formation energy computations"

Hirotoshi Hirai* and Ryosuke Jinnouchi

Toyota Central Research and Development Labs., Inc.,

41-1, Yokomichi, Nagakute, Aichi 480-1192, Japan

Figure S1 shows the total energies of the $Ce_{16}Ti_2Zr_{14}O_{64}$ systems for the symmetrically independent combinations of Ti substitutions. This figure indicates that the two substituted Ti atoms are adjacent to each other in the most stable state.

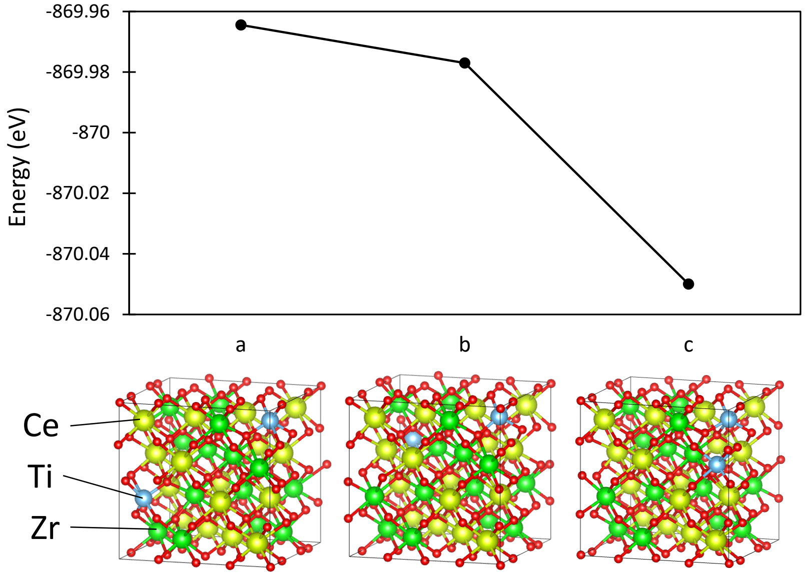

Figure S1: The total energies of the $Ce_{16}Ti_2Zr_{14}O_{64}$ systems for the symmetrically independent combinations of Ti substitutions.

* hirotoshih@mosk.tytlabs.co.jp
